# Community Priority setting for Fetal Alcohol Spectrum Disorder Research in Australia

**DOI:** 10.23889/ijpds.v5i3.1359

**Published:** 2020-12-10

**Authors:** A Finlay-Jones, M Symons, W Tsang, R Mullan, H Jones, A McKenzie, Reibel, L Cannon, B Birda, N Reynolds, P Sargent, H Gailes, D Mayers, EJ Elliott, C Bower

**Affiliations:** 1 Telethon Kids Institute, NHMRC FASD Research Australia Centre of Research Excellence, Nedlands, Western Australia, Australia; 2 Faculty of Medicine and Health and Discipline of Child and Adolescent Health, University of Sydney, Sydney, New South Wales, Australia; 3 Ngangk Yira Research Centre for Aboriginal Health and Social Equity, Murdoch University, Perth, Western Australia, Australia; 4 Faculty of Medicine and Health and Discipline of Child and Adolescent Health, University of Sydney, Sydney, New South Wales, Australia; + Community Member

## Abstract

**Introduction:**

Fetal Alcohol Spectrum Disorder (FASD) is a neurodevelopmental disorder caused by prenatal alcohol exposure (PAE). FASD research is a rapidly growing field that crosses multiple disciplines. To ensure research is relevant and meaningful for people living with FASD, their families, and the broader public there is a need to engage community members in setting priorities for research.

**Objectives:**

Our primary objective was to formally identify the views of people living with FASD, their parents/caregivers, service providers, and the general community on the research priorities for FASD and alcohol use in pregnancy in Australia. Our secondary objective was to provide an overview of current research in the highest priority areas identified.

**Methods:**

The approach for this study involved two community surveys and a consensus workshop, followed by a rapid literature review. Survey responses (n = 146) were collected and grouped using qualitative thematic analysis. The themes identified were then ranked in a second survey (n = 45). The 22 highest ranked themes were considered in a workshop with 21 community members, and consensus on the top ten priority areas was sought. The priority areas were grouped into conceptually similar topics and rapid literature reviews were undertaken on each.

**Results:**

A diverse range of priorities was identified within key areas of prevention, diagnosis, and therapy. On request from participants, separate priority lists were developed by Aboriginal and non-Aboriginal participants.

**Conclusion:**

There is need for a national network of researchers to take forward the research commenced by the Centre of Research Excellence, FASD Research Australia, in addressing community priorities.

**Key Words:**

Community, priorities, FASD, rapid review, Australia

## Introduction

Fetal Alcohol Spectrum Disorder (FASD) is a neurodevelopmental disorder caused by disruption of brain development due to prenatal alcohol exposure (PAE). FASD is characterized by a range of severe, lifelong neurodevelopmental impairments. In Australia, the diagnosis of FASD requires PAE and severe impairment in at least three of ten functional domains [1]. A systematic review and meta-analysis of the prevalence of FASD in the general population in the Western Pacific region (based on two studies, both from Australia) reported a prevalence of 6.7 per 1000, similar to the overall global prevalence of 7.7 per 1000, and the prevalence for the Americas (8.8 per 1000) [2]. Studies in Australia have identified higher rates of FASD in Aboriginal compared with non-Aboriginal children [3-6]. However, FASD prevalence is acknowledged to be underestimated in Australia, due to limitations in diagnosis and reporting [3]. FASD is associated with multiple comorbidities [7] and adverse outcomes include disengagement from school and the workforce and contact with the justice system [8]. The multi-sector impact of FASD is reflected in substantial economic costs: the estimated mean annual cost per child with FASD, based on studies in the US, Canada, Sweden and New Zealand, was US$22,810 [9]. There are no corresponding published estimates for Australia.

The harms caused by PAE are of major public health concern in Australia. Government responses include government inquiries [10], a national FASD Strategic Action Plan 2018-2028 [11], national guidelines for alcohol use [12] and mandating of pregnancy warning labels on alcohol [13]. Australian and State/Territory governments and the National Health and Medical Research Council (NHMRC) have funded several initiatives, including prevalence studies in remote communities [6, 14] and youth detention [8], the development and dissemination of the Australian Guide to the Diagnosis of FASD [1], the national FASD Register [15] and Hub (www.fasdhub.org), and the establishment of diagnostic and management clinics. Consumers and community members have been involved in these and other initiatives, but consumer input has usually followed receipt of funding, so that the FASD research agenda in Australia has been mostly influenced by researchers, clinicians, and funders. To date, there has been no formal attempt to identify the views of parents, caregivers, or the general community on the research priorities for FASD.

Following establishment of the NHMRC-funded Centre of Research Excellence in FASD (FASD Research Australia), a national consumer and community reference group was formed. In collaboration with the Western Australian Consumer and Community Health Research Network (CCHRN), a national priority-setting partnership was formed to establish community priorities for research, based on an abridged model of the James Lind Alliance (JLA) priority setting partnership (PSP). The JLA PSP process has established guidelines and a framework for bringing together researchers, consumers and community members and clinicians to identify unanswered questions (“uncertainties”) in a particular area [16]. Once identified, uncertainties are prioritised so that researchers and funding bodies can be guided by consumer opinion when allocating research time and investment. This paper provides an overview of the process and outcomes of the adapted version of the PSP, reported in detail elsewhere [17]. We also report results of a rapid literature review undertaken to synthesise evidence related to the priorities identified.

## Methods

**Figure 1: Overview of the steps taken in the priority-setting process.**
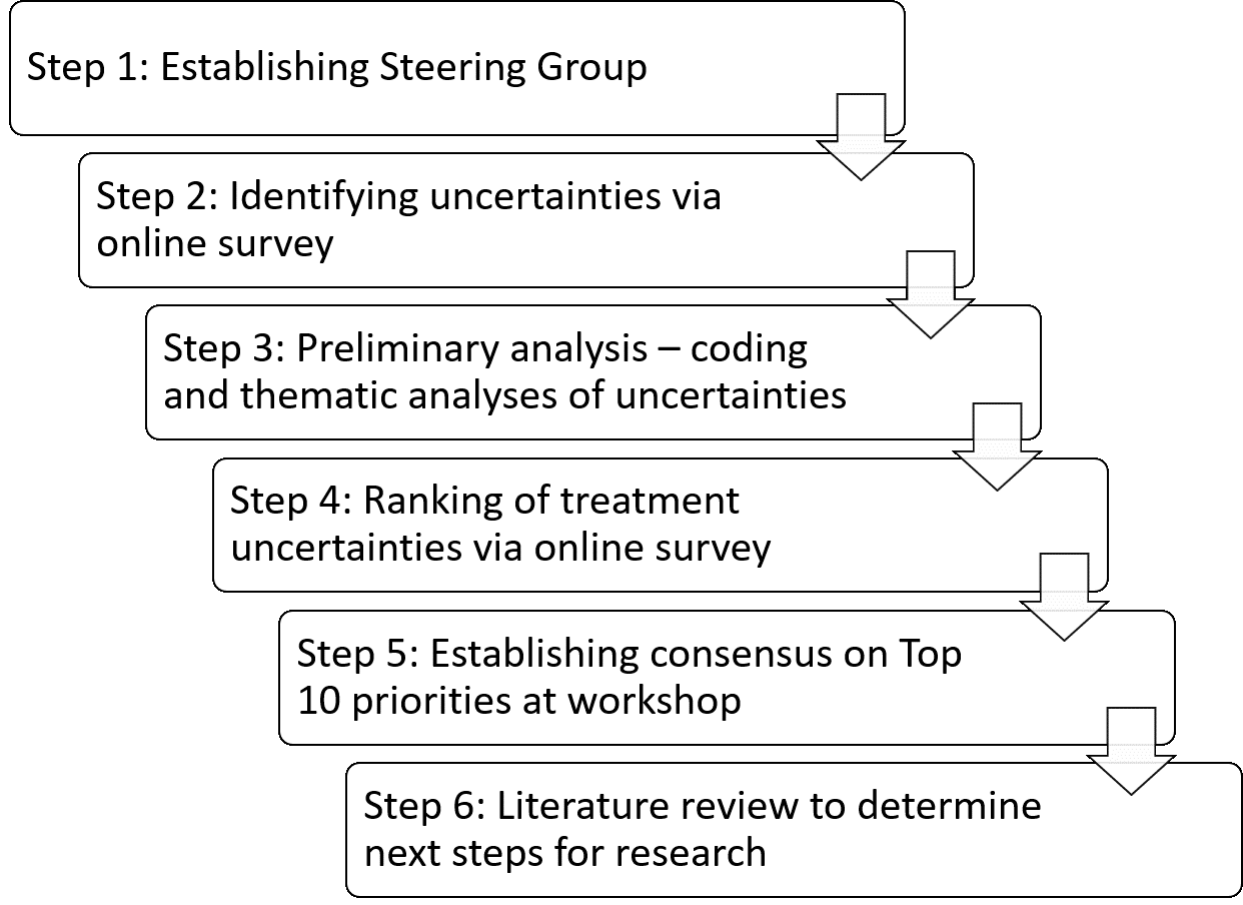


The approach consisted of six steps as shown in Figure 1. Data collection involved two online surveys (Steps 2 and 4) and a consensus workshop (Step 5). While not usually part of the JLA Priority-Setting protocol, a sixth step, involving a rapid literature review, was added to guide future research on FASD in Australia. 

### Step 1: Establishing steering group

The first step was to establish a Steering Group to guide the project activities. The Steering Group consisted of four researchers, two members of the Consumer and Community Health Research Network, and five community members.

### Step 2: Identifying uncertainties

An online survey was developed to elicit questions that community members (including families living with FASD, FASD service providers, and members of the general public) would like addressed through research. The survey and supporting documentation were developed under the guidance of a member of the JLA, based on a process trialed previously by members of our research group. They were then tested and refined by members of the project team, including community, non-clinical and non-research members. The survey comprised four open-ended questions: (1) What questions or concerns do you have about the consumption of alcohol during pregnancy (e.g. how can we encourage family and friends to be supportive?); (2) What questions do you have about the diagnosis of FASD (e.g. how can we get more doctors trained in diagnosing FASD?); (3) What questions do you have about the treatment for FASD (e.g. Why don’t teachers in Australia know more about FASD?); (4) Do you have any other comments (about anything else regarding FASD)?

A snowball sampling strategy was used to distribute the survey to community and professional networks, including the CCHRN’s Involvement Network, FASD Research Australia Community Reference Group’s community networks and FASD service providers. The survey was also promoted through the CCHRN and Telethon Kids Institute social media channels. 

### Step 3: Preliminary analysis

Survey responses were reviewed, checked and verified as true uncertainties, then the resulting list of uncertainties were qualitatively coded by members of the project team using thematic analysis. The final selection of themes was determined by project team consensus. Community members of the project team reviewed the themes prior to Step 4. 

### Step 4: Ranking of major themes

Themes from Step 1 were used to develop an online survey to rank research priorities. The ranking survey was emailed to all respondents to the first survey who had consented to being contacted, and to the broader networks who distributed the first survey. Participants ranked themes in order of importance and individual rankings were combined to provide an overall rank score for each theme. 

### Step 5: Consensus workshop

The 22 themes were presented to a consensus workshop comprising participants recruited from those who completed Steps 1 and 3 and the broader community. The workshop brought together community members with an interest in PAE (n=8); caregivers of people with FASD and people with lived experience of FASD (n=8); and people working in service provision (n=5). Part 1 of the workshop involved review and small group discussion of the 22 themes. To ensure a mix of community members’ interests, participants were designated a table upon arrival. All participants were encouraged to discuss every theme and identify which they felt most and least strongly about.

In Part 2 of the workshop, table facilitators summarised key points from group discussions and shared them with the whole workshop group. Much of the small group discussion was based on whether specific themes should be combined, or new themes established. Members who felt strongly about combining themes or establishing a new theme were invited to present their reasoning to the whole group. A whole group vote determined the final decision.

In Part 3 of the workshop, facilitators from three of four tables integrated the three ranked lists into a single list. There was significant discussion about the need for the process to reflect the needs of Aboriginal people, which were different to those of non-Aboriginal people, and the importance of understanding the impact of intergenerational trauma on Aboriginal people. Following this discussion, it was agreed that rankings from the Aboriginal participants’ table would be captured separately, to ensure their priorities were given appropriate consideration. The workshop participants and facilitators reported being satisfied when consensus was reached and there was shared understanding of the similarities and differences between the two lists. The outcome of having separate lists for Aboriginal and non-Aboriginal priorities mirrors that of a PSP for Family and Domestic violence run by members of our research group [18].

Part 4 of the workshop involved establishing consensus on the final rank of priorities. The themes were displayed on cards on the floor in rank order and the groups were invited to reflect on these priorities. The ranking was discussed by the entire group, with the aim of agreeing on the top ten priorities. The workshop facilitator chaired the discussion to ensure no individual participant dominated the decision-making. If consensus could not be reached by discussion, decisions were put to a vote. Further details on the workshop can be found in the Report on the project [17].

### Step 6: Rapid Review of the Literature

A rapid review of each of the top 10 priorities was conducted to identify relevant studies. We searched for the most recent systematic reviews or meta-analyses for each priority and the most recent individual studies, focusing on studies from Australia where available. Prior to conducting the reviews, priority areas were grouped into conceptually similar topics.

## Results

### Steps 2 and 3: Community survey and coding of responses

A total of 146 community members responded to the first survey: 62% were interested in FASD prevention; 20% worked for an organisation providing services or supporting people with FASD or their carers; and 18% supported someone living with FASD. Participants were aged 18-39 years (41.7%), 40-64 years (52.7%) or 65+(5.4%), 94% were female and 7.4% identified as Aboriginal or Torres Strait Islander. There were 128 responses about PAE, 123 about diagnosis and treatment, and 103 to the item ‘Any other comments about FASD?’. Following coding, 29 themes emerged. Each theme comprised a main question or statement, with one or more examples taken directly from survey responses to provide context (Table 1).

### Step 4: Ranking of major themes

There were 45 responses to the ranking survey. We intended to take the top 20 themes for presentation at the workshops, but as the scores for themes ranked 20, 21 and 22 were similar, we took 22 themes to Step 4. Further details on the themes are available in the detailed report of methods [17].

### Step 5: Consensus Workshop 

Workshop discussions amongst 21 community members resulted in combination of some themes and introduction of some new themes to better capture the agreed intent. The final top 10 priorities for Aboriginal and non-Aboriginal participants are shown in Table 2.

### Step 6: Rapid Review on Each Topic

For the purposes of the rapid review, priorities were clustered into three major topics shown in circles in Figure 2. This diagram also illustrates which major topics the priorities were most aligned with (priorities that related to more than one topic are indicated at the intersection of the circles). Intergenerational trauma, while only identified as a priority for Aboriginal participants, was considered to run across all topics for the purposes of organising the literature.

### Results of rapid review according to major topic

#### Understanding Influences on Prenatal Alcohol Exposure

*Understanding determinants of prenatal alcohol exposure.* Australian data indicate 50-60% of Australian women use alcohol at some point in their pregnancy [19-21]. Insight into the determinants of PAE is key to developing prevention initiatives and providing women with appropriate support. Population-based studies in Australia [22-24] and North America [25] have identified PAE as more common among women who are older, of higher socioeconomic status, and with a higher level of education, despite the latter group being more knowledgeable about the risks of PAE [26]. PAE is also more likely among women who smoke and who have not actively planned their pregnancy [20]. To date, no Australian research has captured the determinants of PAE in a way that enables differentiation of subgroups (e.g. attitudes and beliefs among more educated versus less educated women) and across different time points in pregnancy (for example, motivation to abstain from alcohol use pre-pregnancy awareness, versus post-pregnancy awareness).

Most women who use alcohol in pregnancy do so before being aware that they are pregnant, and reduce their use once pregnancy is confirmed [16, 21]. As highlighted by McCormack et al. [20], drinking alcohol after pregnancy awareness is a conceptually different behaviour to drinking before pregnancy recognition, and may have different determinants. Given that approximately 50% of pregnancies in Australia are unplanned [27], more research is required to understand how to prevent alcohol exposure in unplanned pregnancies prior to pregnancy recognition (e.g. via more effective contraception use, reduction in alcohol use, or both), as well as how to prevent exposure in planned pregnancies prior to pregnancy recognition.

*Changing societal views and beliefs about PAE.* Workshop discussions on this topic highlighted that FASD prevention efforts should encompass pregnant women’s partners and children (amongst others) given that PAE occurs in the context of alcohol use and behaviour of partners, family members, and friends [28, 29]. These findings align with results of a recent systematic review that identified father’s alcohol use before or during pregnancy as a key influence on mother’s alcohol use [30]. Motivational text messages presented in the voice of a child may be one way to increase the awareness of fathers about the dangers of alcohol use during pregnancy [31]. In Australia, policies which may be effective in reducing population alcohol consumption, such as restriction of supply or access (e.g. increase in price via taxation)[32] have been implemented in a piecemeal fashion or not at all.

*Understanding the impact of intergenerational trauma.* Health behaviours, including alcohol use, among Aboriginal Australians are severely impacted by colonisation and the resulting economic and cultural exclusion and loss of land [33]. A range of complex social and environmental factors can lead to higher levels of alcohol consumption including stress, racism, social inequalities, lack of meaningful work and poor early life experiences [33, 34]. It is plausible that these issues underpin the high rate of FASD reported in some Aboriginal populations [3, 5, 6] although, importantly, FASD is not limited to these populations. Given Australia’s history of colonisation, addressing the effects of intergenerational trauma is likely to be important for reducing PAE among Aboriginal communities. Work in South Africa has found that women with trauma exposure are likely to continue using alcohol in pregnancy at pre-pregnancy levels [35].

#### Preventing PAE and Promoting Maternal and Child Health

*Development of a national public health campaign.* Although this theme is conceptually linked to changing societal views and beliefs, workshop members believed it was important to consider it independently. The Australian Government recently announced $25 million to be provided to the Foundation for Alcohol Research & Education for a national awareness campaign. To maximise effectiveness there are fundamental research questions: (1) What are the key constructs that should be targeted by a national public health campaign?; (2) How should messages be framed?; and (3) How should the campaign be delivered? It is important these questions be considered using evidence of effectiveness of public health campaigns targeting PAE. Previous studies of prevention messaging in Australia found ‘threat appeal’ was more effective than a ‘self-efficacy’ message for increasing women’s intentions to abstain from PAE [36]. The authors recommended that combining threat and self-efficacy messaging in future mass-media campaigns would be most effective, through provoking both positive and negative emotional responses without unintended stigma or defensive consequences [36].

A recent systematic review of mass media campaigns to reduce alcohol use and alcohol-related harms included 24 studies [37]. Four were mass media campaigns targeting reduction of PAE. Three of these were US studies [38-40] and one Canadian [41]. No study assessed impact on alcohol use behaviour. Awopetu and colleagues [38] found a significant post-campaign increase in treatment/help-seeking phone calls about fetal alcohol syndrome (FAS) compared to pre-campaign. The other three studies targeted knowledge/awareness of the risks of PAE. Lowe and colleagues [40] found a significant increase in knowledge of the risks of PAE in the campaign-exposed group relative to a control group, but results of the uncontrolled studies were mixed [41]. Overall, the review [37] showed weak evidence for alcohol campaign recall and impact on knowledge, attitudes and beliefs and no evidence of reduced alcohol use. There is a need for research to be embedded into any public health campaign to address implementation questions prior to rollout and evaluate effectiveness afterwards.

*Education in high school about the risks of PAE* Despite mixed findings, a recent systematic review found sufficient evidence to support the use of school-based universal prevention strategies to reduce alcohol use in Australia. However, the impact of such programs on PAE is unclear [32]. The current literature search returned three US-based prevention education studies. One intervention [42] was specifically designed for Native American adolescents, while two [43, 44] were peer-delivered programs to educate students about FAS/fetal alcohol effects (FAE). All three studies revealed most students initially knew little about FAS/FAE; and knowledge about PAE and FAS/FAE improved markedly after the program. Two of these studies also measured attitudes, with one finding an increase in positive attitudes [42]; and the other finding no effects [43]. Given that improving knowledge and/or attitudes about a specific health behaviour may not change behaviour [42, 43] there is a need for further research examining the impact of such programs on alcohol use in pregnancy.

Together, the reviewed studies suggest initially targeting grades 6-8 and continuing education through high school—with age-appropriate content, language and presentation style [42, 44]—and prevention programs that include skill-building activities based on health education principles [43]. The recommendation for age-specific programs corresponds with a recent meta-analysis regarding school-based substance-use prevention programs [45]. Although most students benefit from self-control training, problem-solving skills training and cognitive behavioural therapy techniques, programs aimed at middle adolescents (grades 8 and 9) had the least effect [45]. The meta-analysis emphasised that effective school-based prevention programs should consider adolescents’ physical and social environments and their biological, emotional, cognitive and social developmental stages.

*Promoting maternal health during pregnancy.* The need to support maternal health during pregnancy was identified as the third priority for Aboriginal participants in our study. In Australia, there are distinct disparities in pregnancy, labour, and neonatal outcomes between Aboriginal and non-Aboriginal people. For example, preterm birth, stillbirth, and small for gestational age births are substantially higher among Aboriginal Australians;[46] and can be associated with PAE [47, 48]. In addition to the direct impact of colonisation on Aboriginal health outcomes, several factors impede provision of appropriate antenatal care, including differences in culture and language and poorer access to alcohol and other drug services in regional and remote areas [49]. To increase the likelihood of PAE prevention in Aboriginal communities, community consultation, provision of culturally safe services, and integration of culture into alcohol treatment are recommended [50].

A systematic review returned 23 studies of Aboriginal and Torres Strait Islander maternal and child wellbeing programs [51]. Of these, 52% reported programs delivered within Aboriginal Community Controlled Health Organisations - a factor considered fundamental to improved outcomes. Positive outcomes, including increased antenatal attendance and higher infant birth weights, were reported in some intervention studies, however issues with study quality precluded the authors from making firm conclusions about the evidence [51]. There is a need for further research that integrates community-led programs in the context of trials designed to establish evidence of effectiveness and cost-effectiveness.

*Role of immediate, extended family and/or community and being good role models to support women.* There is evidence to support the effectiveness of family-based approaches to reducing alcohol consumption in the general population [52]. Exciting new areas of research and translation are adapting these approaches specifically for pregnancy, including Aboriginal-led development of approaches that incorporate the whole community to support women. Although there is currently little evidence of effectiveness of community-based approaches [32], emerging research demonstrates their feasibility and acceptability when adapted for Aboriginal communities [53, 54]. Additionally, there is evidence that combinations of family, school, and community approaches can be effective in reducing alcohol consumption generally [32].

*Identifying the best/most effective support to prevent PAE.* Australian women who drink alcohol in pregnancy are not a homogenous group and may require different types of support to reduce PAE, ranging from primary prevention to specialised antenatal care. For women at high-risk of drinking alcohol, the Parent-Child Assistance Program (PCAP), which provides three years outreach of case-manager support, has proven cost-effective in both the US and Canada for reducing PAE [55, 56]. More generally, Screening, Brief Intervention and Referral to Treatment (SBIRT) approaches have been recommended and evaluated, however the evidence for these approaches is equivocal [57]. Antenatal care models incorporating caseload midwifery and continuity of care are also recommended as part of a multi-level prevention approach [58].

*Health professionals’ knowledge about alcohol in pregnancy and provision of non-judgmental pregnancy support.* Australian research demonstrates that among health professionals caring for pregnant women, there is infrequent provision of advice consistent with national guidelines on alcohol use [59-62], although this improves following educational interventions [63, 64]. Interestingly, recent work has revealed that higher levels of health literacy regarding PAE and FASD, and stronger endorsement of prevention messaging is also associated with greater stigma toward those who use alcohol in pregnancy [65]. These findings reveal the need to balance clear messaging around PAE with a de-stigmatising, trauma-informed approach that considers the multiple influences on PAE [66]. In Australia, a randomised step-wedged cluster trial is underway and will provide the first evidence of effectiveness of implementation strategies directed to health professionals in antenatal care to decrease PAE [67].

#### Improving FASD Diagnosis and Management

*Training health professionals in FASD diagnosis and management/support.* Training in use of the Australian Guide to the Diagnosis of FASD has been widespread amongst clinicians [1, 68]. However, because of the wide range of severe impairments in FASD and secondary effects on schooling, employment, mental health and contact with the justice system, interdisciplinary and multi-sector capacity building in the workforce (within and beyond health) is required to fully support FASD screening, diagnosis, referral, and management. Recent Australian research has demonstrated this need in the justice system [69], and documented barriers to implementation of clinical guidelines for preventing PAE in antenatal services. Given that screening for alcohol use is central to prevention and diagnosis, further implementation research is required to overcome these barriers [62].

*Supporting parents and families living with FASD* Caregivers of children with FASD frequently report high levels of stress, feelings of isolation and a strain on their child-caregiver relationship [70, 71]. Although the need for self-care is recognised by caregivers, limited time, resources, and social support may undermine caregiver capacity to engage in self-care behaviour [72]. Additionally, it has been documented that while early intervention for FASD may meet the child’s cognitive, physical, communication or adaptive needs, the broader needs of the family and social-emotional needs of the child are often unmet. Families also express the need for more support following diagnosis and navigating transition through different life phases and support systems [73, 74].

Given the range of strengths and difficulties individuals living with FASD and their families may experience, the utility of one-size-fits-all support approaches is likely to be limited. Parent-mediated interventions that focus on supporting caregivers to manage their child’s behaviour and improve their caregiver skills (e.g. self-efficacy; self-care; educating caregivers about FASD, behavioural learning principles and advocacy) are promising [75]. However, despite FASD being a life-long condition and caregiver mental health being a key contributor to caregiver and family wellbeing, research-based interventions aimed at adolescents and adults living with FASD, and caregiver mental health are limited [71, 75]. A key question for research, practice, and policy is how to establish systems of care that are responsive to the unique needs of each family and individual living with FASD and adaptable to changing demands of transitions through different life stages.

## Discussion

We aimed to establish research priorities regarding PAE and FASD among members of the Australian public using an abridged version of the JLA PSP process. Although our approach was limited by the small number of participants at each step, this is the first time that a structured approach to identifying community priorities on this topic has been undertaken. Accordingly, our findings are valuable in establishing community perspectives to guide future FASD research and translation activities. A key recommendation is the need for further research to provide evidence both for effective prevention of PAE and FASD and interventions to improve outcomes for those living with FASD. The current evidence for effective interventions is weak, despite a range of prevention activities currently being undertaken. Furthermore, approaches must be tailored to the needs and preferences of specific populations.

Priorities identified regarding FASD diagnosis focused on provision of additional training to help health professionals to recognise and provide diagnostic services. Many resources have been developed and distributed in Australia in recent years and diagnostic services are becoming more available, and both diagnostic training and resources should be evaluated. There needs to be a commensurate level of management services available for those diagnosed with FASD, especially in rural and remote areas and this could be the topic of future health service research. Support for those with FASD and their carers was identified as a priority, and it appears that this need is not currently met in Australia. The rapid review of the literature revealed that while there is preliminary evidence of efficacy of a range of interventions for FASD, there is currently no “gold-standard” approach. This reflects the heterogeneity in clinical presentation of FASD, which necessitates a more personalised approach to intervention. Key directions for future research include development and testing of interventions that are tailored to the functional needs and resources of the individual and their family.

It is perhaps not surprising that, of the top 10 priorities for Aboriginal and non-Aboriginal community members, there were seven in common. Importantly though, priorities for Aboriginal community members highlight the role of family and community in supporting pregnant women and people living with FASD, the impact of intergenerational trauma on alcohol use and neurodevelopmental outcomes, and the need for a more holistic approach to supporting pregnancy health. For non-Aboriginal community members, there was more emphasis on the diagnosis and management of FASD, and the role of the healthcare system. These differences highlight the importance of Aboriginal leadership in research involving Aboriginal communities, as well as the role of Aboriginal-led organisations in supporting the translation of research into practice.

Given that many priorities that emerged from the current project align with those expressed regarding neurodevelopmental disorders more broadly [76], consideration may be given to trans-diagnostic or cross-syndrome approaches to research and translation. Such approaches focus on promoting healthy development and optimising functional outcomes across the spectrum of neurodevelopmental disability, as well as among those with subclinical neurodevelopmental vulnerability [77]. Because frequent co-morbidities of FASD include other neurodevelopmental disorders, physical illnesses, and mental ill-health, there is also a need for research on integrated care pathways for affected individuals and families. As highlighted in Priority 3 by Aboriginal workshop participants’ priority of “mothers staying healthy during pregnancy,” it is also important to consider alcohol exposure as part of a comprehensive approach to promoting health prior to conception and during pregnancy.

A national network of researchers is required to take forward the work of FASD Research Australia in addressing the research priorities on FASD and PAE identified by the people with lived experience of FASD and the general public. This will require a coordinated and collaborative approach.

**Table 1: Themes Identified from online survey by question ordered by the highest number of responses in each area:** 

Theme (n = 146)
Questions or Concerns about consumption of alcohol during pregnancy

Is there a safe level of alcohol use during pregnancy?
What are the harms caused by drinking alcohol while pregnant?
Are there certain periods within pregnancy that drinking alcohol causes higher risk of FASD?
Pregnancy planning and drinking prior to pregnancy recognition
Doctors and/or other health professional's knowledge
The need for education/awareness raising in communities and schools
Involvement of men

Questions about the diagnosis of FASD

Who/where/how are FASD diagnoses made?
How much do health professionals know?
What is the prevalence of FASD?
What is the optimal diagnosis age?
How does it compare to ADHD, autism and other conditions? Is there misdiagnosis?
What are the signs and symptoms of FASD?
What are the outcomes/benefits to diagnosis?

Questions about treatment for FASD

Is there a treatment or cure?
What is the optimal age for intervention and support?
What is the best/most effective support for those with FASD?
What support is there for accessing and financial support for treatment?
What outcomes can be expected from treatment?
How can schools support students with FASD?
What support is there for parents/families?

Any other comments about FASD

More awareness raising needed
Risks of alcohol consumption are not promoted enough in health workers
More/Correct diagnosis needed
Need more clinics/services/supports

**Table 2: Top 10 Research Priorities from Consensus Workshop for Aboriginal and Non-Aboriginal Community Members. For each research priority on the Aboriginal Community Members list, the figure in brackets represents how the priority ranked on the non-Aboriginal Community Members list, and vice versa.** NA – This was not a top 10 priority for the other group of community members

	Aboriginal Community Members	Non-Aboriginal Community Members
1	National public health campaign/education (3)	Changing societal views and beliefs about PAE (8)
2	Education during high school about the risks of PAE (4)	Doctors’ and/or other health professionals’ knowledge about PAE (9)
3	Mothers staying healthy during pregnancy (NA)	National public health campaign/education (1)
4	Role of immediate, extended family and/or community and being good role models to support women (NA)	Education during high school about the risks of PAE (2)
5	Providing non-judgmental pregnancy support (9)	Training in FASD diagnosis (NA)
6	Intergenerational trauma (NA)	Training in FASD management/support (NA)
7	Understanding determinants of PAE (7)	Understanding determinants of PAE (7)
8	Changing societal views and beliefs about PAE (1)	Identifying the best/most effective support for PAE (10)
9	Doctors’ and/or other health professionals’ knowledge about PAE (2)	Providing non-judgmental pregnancy support (5)
10	Identifying the best/most effective support for PAE (8)	Supporting families and parents living with FASD (NA)

**Figure 2: Priorities mapped onto three major topics. Rankings for each priority are presented as (Aboriginal Community Ranking/non-Aboriginal Community Ranking):**
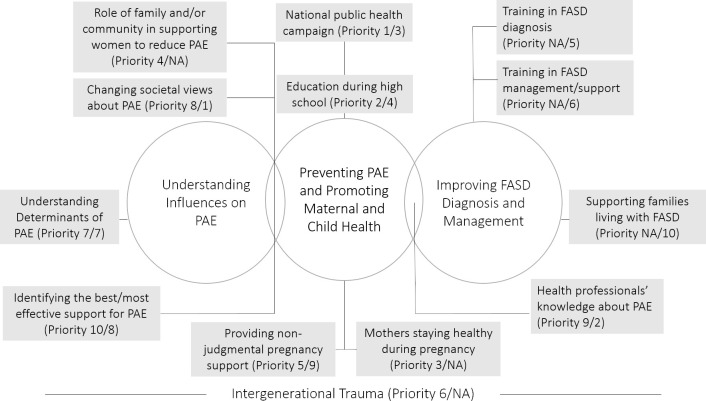


## Acknowledgments

The authors would like to acknowledge the input of all community members who took part in this priority setting process, as well as Sally Crowe who provided oversight for the approach used.

## Ethics statement

This project was granted ethical approval from the University of Western Australia Human Research Ethics Committee (#RA/4/1/9240). All participants gave informed consent before participating in the study.
